# A Rapid Method for Accurately Determining Lipid Nanoparticle Size Using Nano-Flow Cytometry

**DOI:** 10.7171/001c.163225

**Published:** 2026-06-30

**Authors:** Brittany Rein, Christiano Marconi, Daniil Shabashvilli, Paul Chipman, Mary Gragg, Elias Sayour, Anderson Silva, Mariza Miranda, Steven Madore

**Affiliations:** 1 ICBR University of Florida; 2 Department of Neurosurgery University of Florida

**Keywords:** nano-flow cytometry, CryoTEM, lipid nanoparticles

## Abstract

Lipid nanoparticles (LNPs) are small particles composed of lipids that can be used to encapsulate and deliver therapeutic substances like mRNA or other drugs to specific cells or tissues. LNPs are specifically designed to protect the payload from degradation, enhance payload stability, and improve delivery to target cells and tissues. When developing LNPs for nucleic acid-based therapeutics, there are two characteristics that are often analyzed to determine the quality of the formulations: size distribution and loading efficiency. Various techniques are used to determine the size of nanoparticles, with each providing different details and having specific advantages and limitations. In this study, we evaluated the use of nanoparticle tracking analysis, dynamic light scattering, and two different nano cytometers to determine LNP size. We compared the results obtained from these four methods to the particle size determined using cryogenic transmission electron microscopy. Our findings showed that nano-flow cytometry instrumentation, like NanoFCM and CytoFLEX nano, offers a robust and accurate method for sizing LNPs with measurements in high concordance to those obtained by conventional cryogenic transmission electron microscopy.

## Introduction

**Figure 1.**
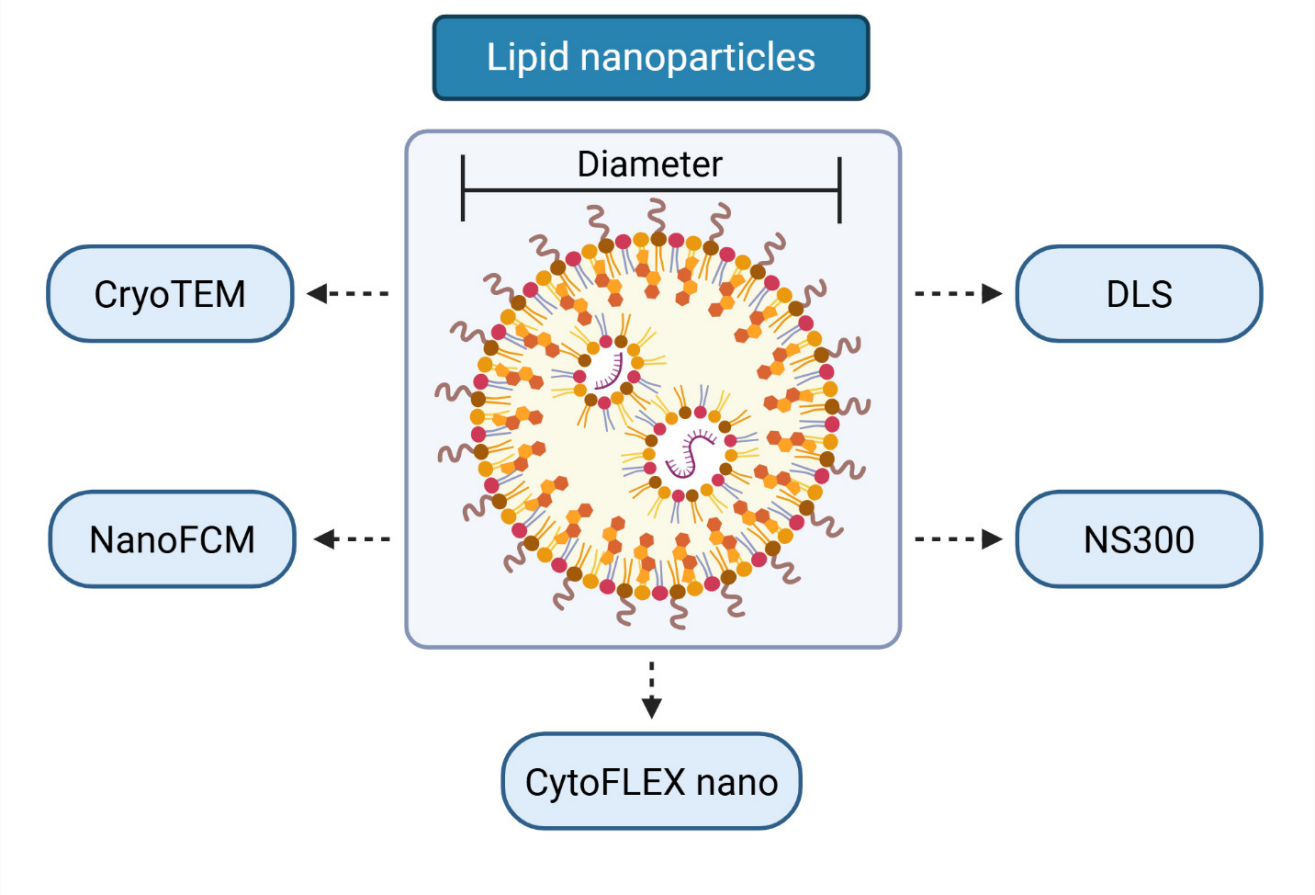
Graphical Abstract.** Different techniques to measure lipid nanoparticle size.

Lipid nanoparticles (LNPs) are small spherical particles composed primarily of lipids, typically ranging from 20 to 1000 nm in diameter.^1^ They have emerged as a promising platform for delivering nucleic acid-based therapeutics, including mRNA vaccines and gene editing tools. The size of LNPs influence biodistribution, cellular uptake, and therapeutic efficacy; therefore, accurate characterization of LNP size is essential.^2^ (See Figure 1 for different techniques to measure lipid nanoparticle size).

Cryogenic transmission electron microscopy (cryo-TEM) is widely regarded as the reference method for direct physical size measurement of LNP morphology and size. Cryo-TEM can accurately measure the true size of particles by looking at electron density.^3^ However, cryo-TEM is technically demanding, time-intensive, and low-throughput, requiring specialized equipment and expertise that may not be readily accessible in all laboratory settings. Nanoparticle tracking analysis (NTA) and dynamic light scattering (DLS) are also commonly used methods of characterizing LNP size and concentration. These techniques measure hydrodynamic diameter and are highly dependent on temperature and buffering conditions. They are also limited by user input factors, such as brightness, focus, and speed settings for the NTA and attenuator level and scatter angle for DLS, respectively. These factors may lead to inconsistent results due to subjectivity in user input.^4^

Alternative methods that balance accuracy and efficiency with ease of use and affordability are needed to overcome these limitations. One such platform, nanoparticle flow cytometry (NanoFCM), has recently gained attention as a promising new tool for high-resolution nanoparticle size analysis. NanoFCM uses light scattering and fluorescence, which allow for the simultaneous measurement of size and concentration of nanoparticles, including extracellular vesicles, viruses, and LNPs.^5,6^ One key advantage of NanoFCM is that it uses silica nanoparticles as size reference standards. Silica nanoparticles have a refractive index (RI) that closely matches that of biological particles such as extracellular vesicles (~1.40) and viruses (~1.46).^6–8^ This similarity is the primary reason why NanoFCM claims to produce results comparable with TEM measurements using only side-scatter detection. This technique also offers significant improvements in throughput and ease of use.

In this study, we evaluated the performance of the conventional cryo-TEM measurements for LNP sizing compared to results obtained using NanoFCM, CytoFLEX nano, DLS, and NTA. Our results indicate that NanoFCM serves as a rapid, reliable, and accessible alternative for cryo-TEM, providing similar size-resolution capabilities while requiring far less effort to implement.

## Materials and Methods

### LNP Preparation

Lipid nanoparticles (LNPs) were fabricated according to the LNP fabrication method described by Grippin et al (2025).^9^

### NanoFCM Analysis

The NanoFCM was set up following the manufacturer’s instructions (NanoFCM Co., Ltd., Nottingham, United Kingdom). Briefly, the instrument was calibrated before each use with 250-nm silica nanospheres (QC Beads, Cat. #S08210, NanoFCM Co., Ltd.) diluted 100-fold in MilliQ water to determine the standard particle concentration (particles/mL) and sample flow rate (nL/min). Size calibration for small particles was performed using S16 size silica calibration beads of 68, 91, 113, and 155 nm (Cat. #S16M-Exo, NanoFCM Co., Ltd.). Samples were serially diluted at a 1:200,000 ratio in a 1x tromethamine-ethylenediaminetetraacetic acid buffer and were acquired at 1.0 kPa per 1 minute on a NanoFCM (NanoFCM Co., Ltd.) using side-scatter (SSC) triggering with a 488-nm laser using the software’s built-in automatic small signal threshold to gate the nanoparticles from noise as shown in Supplemental Figure 2. Data was acquired in triplicate (minimum of 3000 events per replicate) and processed using NanoFCM analysis software (v2.0). Populations were assessed for mean particle diameter (nm) and concentration (particles·mL⁻¹).

**Figure 2.**
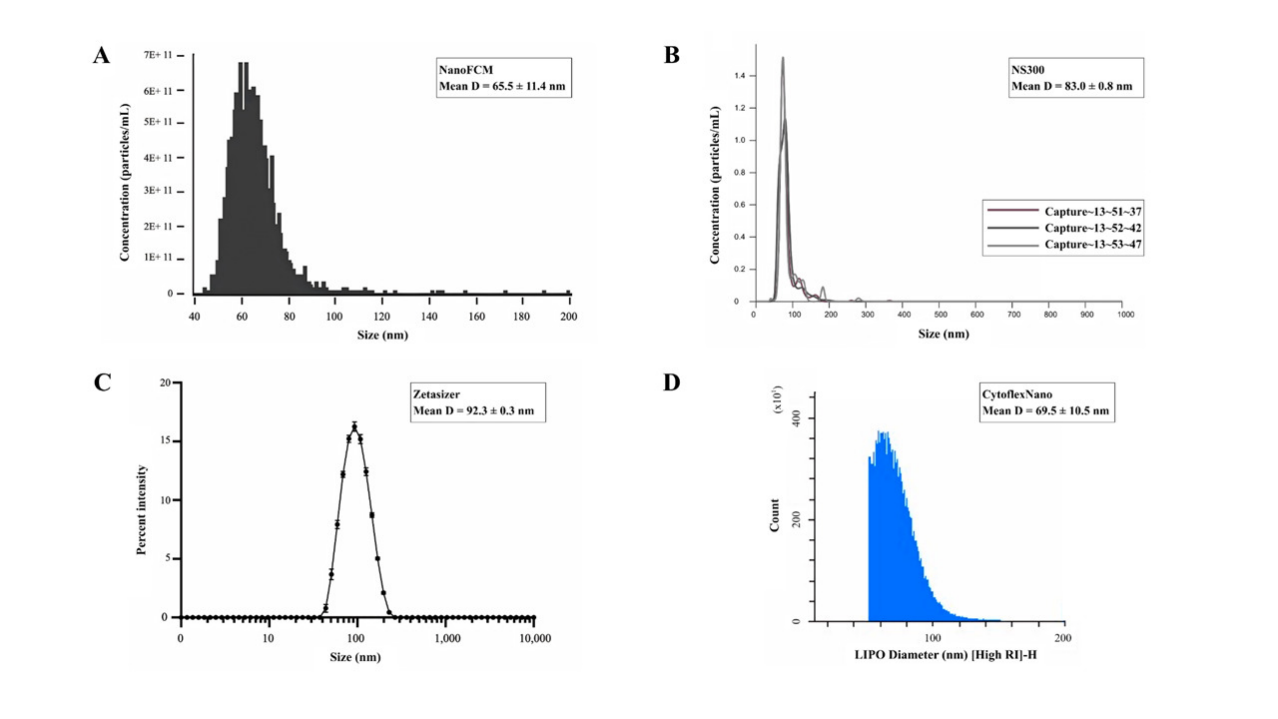
Determination of LNP diameter. At least three aliquots from the same LNP preparation were analyzed by (A) NanoFCM; (B) NanoSight NS300; (C) Zetasizer Nano ZS; and (D) CytoFLEX nano. Mean D represents the average diameter obtained from at least three different aliquots from the same LNP preparation.

### CytoFLEX nano Analysis

Set-up of the CytoFLEX nano (Beckman Coulter, Brea, CA, USA) was as described by Messaggio (2024).^10^ CytoFLEX sheath fluid (Beckman Coulter) was used to perform the runs on the CytoFLEX nano. NIST 100-nm beads were used for size calibration. Unstained LNPs were diluted at a ratio of 1:100,000 in 1x Phosphate Buffered Saline (PBS) and analyzed using a CytoFLEX nano configured for small particle detection. Blank 1x PBS was analyzed as a control. Side scatter was triggered on the 405-nm laser (violet SSC) detection, which has the highest sensitivity for sizing. Data was collected in triplicate, and populations were analyzed using FCMPASS software following the guidance of Welsh and Jones (2021).^11^ The calibration was done on VSSC2 to fit the full range of sizing beads to determine the collection angle. This collection angle was then used on FCMPASS along with the NIST 100-nm size beads.

### Nanoparticle Tracking Analysis (NTA)

Unstained LNPs were diluted at a ratio of 1:50,000 in 1x PBS and analyzed on a NanoSight NS300 (Malvern Panalytical, Malvern, United Kingdom) equipped with a 488-nm laser. Three consecutive 60-second videos were recorded under the following instrument settings: camera level of 15, detection threshold of 5, and syringe pump speed of 35. NIST 100-nm polystyrene beads were diluted at a ratio of 1:25,000 in 1x PBS and run as a control. Three consecutive 60-second videos were recorded under the following instrument settings: camera level of 12, detection threshold of 5, and syringe pump speed of 30. Videos were processed using NTA 3.4 software to determine mean size (nm) and particle concentration (particles·mL⁻¹).

### Dynamic Light Scattering (DLS)

Hydrodynamic diameters were determined using a Zetasizer Nano ZS (Malvern Panalytical). NIST 100-nm polystyrene beads were run as a control. LNP samples and polystyrene beads were diluted until they fell into a rate of 300–600 kcps at an attenuator level of 8 (a ratio of 1:1000 for LNPs and 30:1000 for polystyrene beads in ultrapure water, respectively). Samples were equilibrated at 25°C in disposable polystyrene cuvettes and measured via backscatter sizing in triplicate. Results are reported as the intensity-weighted Z-average diameter ± standard deviation. LNPs were run using the preset liposome RI setting (1.45), while polystyrene beads were run on the preset polystyrene latex bead RI setting (1.59).

### Cryogenic Transmission Electron Microscopy (Cryo-TEM)

Particle size determination was obtained by preparing replicate samples on four separate grids. Cryo-TEM grids were prepared by applying 3 uL of LNP sample onto freshly glow-discharged Quantifoil copper grids (Quantifoil R 2/2 400 mesh), blotting, and vitrifying using a Vitrobot Mark 4 (FEI) at 95% humidity and 4°C. Using a Glacios G2 cryo-TEM equipped with a Falcon 4i camera (Thermo Fisher Scientific Inc., Waltham, MA), four replicate data sets were collected from the grids, each composed of images of four individual 2-µm holes within the carbon support film of the electron microscopy (EM) grids. All LNPs in the holes of the four sets of data were measured using ImageJ software. The diameter and standard deviation were calculated from all collected data.

### Data Analysis

Mean particle size measurements obtained from the same batch of LNPs were compared across five platforms: Zetasizer Nano ZS (DLS), NS300 (NTA), NanoFCM, CytoFLEX nano (after FCMPASS analysis), and Glacios G2 (cryo-TEM). For each platform, size estimates were calculated according to standard instrument-specific reporting conventions: intensity-weighted means for NTA and DLS and means for NanoFCM, CytoFLEX nano, and cryo-TEM. Independent replicate measurements (n = 2–4 per instrument) were used for the analysis. Overall differences among instruments were evaluated using one-way Welch analysis of variance (ANOVA) followed by pairwise Welch’s t-test with Holm correction using cryo-TEM as the reference. Results are reported as mean ± standard deviation with 95% confidence intervals based on independent measurements. Particle concentration measurements were included where applicable.

## Results

### Particle Size Determination

Unstained LNPs analyzed in triplicate by NanoFCM had an average diameter of 65.5 ± 11.4 nm (Figure 2a). The same formulation showed a mean diameter of 69.5 ± 10.5 nm when analyzed using the CytoFLEX nano and processed using FCMPASS software. The NanoSight NS300 reported a mean diameter of 83.0 ± 0.8 nm and a total concentration of 3.75 × 10¹³ particles·mL⁻¹ for the LNPs (Figure 2b). Zetasizer Nano ZS analysis yielded a diameter of 84.6 ± 0.09 nm (Figure 2c). DLS is a measure of the Z-average, which makes the method more susceptible to skewing. Both measurements were larger than those obtained using nano-flow cytometry and cryo-TEM, but they were consistent with the expected bias from light-scattering intensity measurements.^4^

### Particle Morphology and Size Distribution by Cryo-TEM

**Figure 3.**
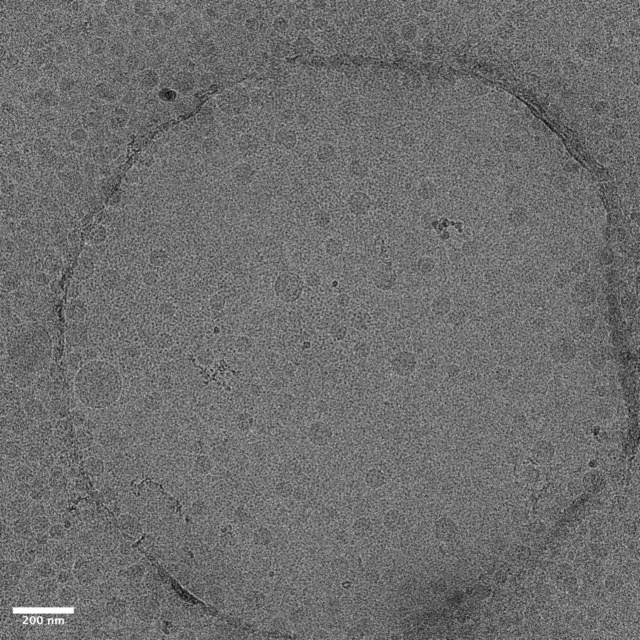
LNP size determination by cryo-TEM. A representative micrograph of one 2-µm hole within the carbon support film with LNPs suspended in a layer of vitreous ice.

LNP imaging was performed using the Glacios G2 cryo-TEM. All LNPs from four sets of replicate grids were measured using ImageJ software, and the diameter and standard deviation were calculated from a total of 686 LNPs. Mean diameter was defined to be 68.9 ±16.3 nm. The majority of LNPs appeared round in the images, which suggest they have a spherical morphology (Figure 3).

## Discussion

This comparative analysis highlights the variability inherent to different nanoparticle sizing methods. While cryo-TEM offers direct visualization and accurate size assessment, nano-flow cytometry techniques proved to be the most comparable optical technique for LNP sizing, particularly in the sub-100 nm range. One-way Welch ANOVA followed by Holm-adjusted post-hoc pairwise comparisons showed that measurements obtained using Zetasizer Nano ZS (DLS) and NanoSight NS300 (NTA) were significantly different from those obtained by cryo-TEM (Table 1). This indicates that NTA and DLS measurements should be interpreted with caution for narrowly distributed nanoparticles due to their tendency to overestimate size and mask minor populations.^4^

**Table 1.** Comparison of mean particle diameter (Mean D) obtained from NanoSight NS300, Zetasizer Nano ZS, NanoFCM, and CytoFLEX nano with Mean D determined using cryo-TEM. Overall differences among instruments were evaluated using a one-way Welch ANOVA, followed by a pairwise Welch’s t-test with Holm correction using cryo-TEM as the reference.

Instrument	Mean D (nm)	Holm-Adjusted *p*-Value
NanoSight NS300 (NTA)	83.0 ± 0.8	0.00079*
Zetasizer Nano ZS (DLS)	92.3 ± 0.3	0.00075*
NanoFCM (Nano-flow cytometry)	65.2 ± 11.4	0.08600
CytoFLEX nano (Nano-flow cytometry)	69.5 ± 10.5	0.92422
Glacios G2 (cryo-TEM)	68.9 ± 16.3	--

*Significantly different from cryo-TEM measurement (*p* < 0.001).

When selecting LNP sizing methods, it is important to consider the most feasible balance between user accessibility, throughput, resolution, and accuracy necessary for the specific application. Among the methods compared, NanoFCM and CytoFLEX nano most accurately matched the size determined using cryo-TEM. Although providing a monodisperse size distribution with consistent concentrations, both NanoSight NS300 and Zetasizer Nano ZS overestimated particle size. We conclude that our comparative analysis validates the use of nano-flow cytometry techniques for accurately determining LNP size and provides a rapid, cost-effective, and high-throughput alternative to cryo-TEM.

### Conflicts of Interest

The authors declare no conflict of interest.

## Supplementary Material

Supplemental 1a

Supplemental 1b

Supplemental 1c

Supplemental 2

Supplemental 3

Supplemental 4

Supplemental 5

## Data Availability

All raw data files including FCS, NFA, NTA, and XSLS files have been deposited in the Genboree NanoFlow Repository App and are publicly available through Team UF ICBR CY.

## References

[1] McMillan C., Druschitz A., Rumbelow S.. (2024). Tailoring lipid nanoparticle dimensions through manufacturing processes. RSC Pharm.

[2] Takechi-Haraya Y. (2025). Cryogenic transmission electron microscopy for the evaluation of lipid-based nanomedicines: principles, applications, and challenges. Biol Pharm Bull.

[3] Hallan S. S., Sguizzato M., Esposito E., Cortesi R. (2021). Challenges in the physical characterization of lipid nanoparticles. Pharmaceutics.

[4] Kaasalainen M., Aseyev V., von Haartman E.. (2017). Size, stability, and porosity of mesoporous nanoparticles characterized with light scattering. Nanoscale Res Lett.

[5] Zhang W., Tian Y., Hu X.. (2018). Light-scattering sizing of single submicron particles by high-sensitivity flow cytometry. Anal Chem.

[6] Tian Y., Xue C., Zhang W.. (2022). Refractive index determination of individual viruses and small extracellular vesicles in aqueous media using nano-flow cytometry. Anal Chem.

[7] Narváez-Narváez D. A., Duarte-Ruiz M., Jiménez-Lozano S.. (2023). Comparative analysis of the physicochemical and biological characteristics of freeze-dried pegylated cationic solid lipid nanoparticles. Pharmaceuticals (Basel).

[8] Nordström R., Zhu L., Härmark J.. (2021). Quantitative cryo-TEM reveals new structural details of Doxil-like pegylated liposomal doxorubicin formulation. Pharmaceutics.

[9] Grippin A. J., Marconi C., Copling S.. (2025). SARS-CoV-2 mRNA vaccines sensitize tumours to immune checkpoint blockade. Nature.

[10] Messaggio F. (2024). A New Approach to Nanoscale Flow Cytometry with the CytoFLEX Nano Analyzer.

[11] Welsh J. A., Jones J. C., Tang V. A. (2020). Fluorescence and light scatter calibration allow comparisons of small Particle Data in standard units across different flow cytometry platforms and detector settings. Cytom A.

