# Supplementary figures and images for: A Rapid Method for Accurately Determining Lipid Nanoparticle Size Using Nano-Flow Cytometry
Source: J Biomol Tech. 2026 Jun 30;37(2):48–53. doi: 10.7171/001c.163225 (PMC13327552; doi:10.7171/001c.163225)

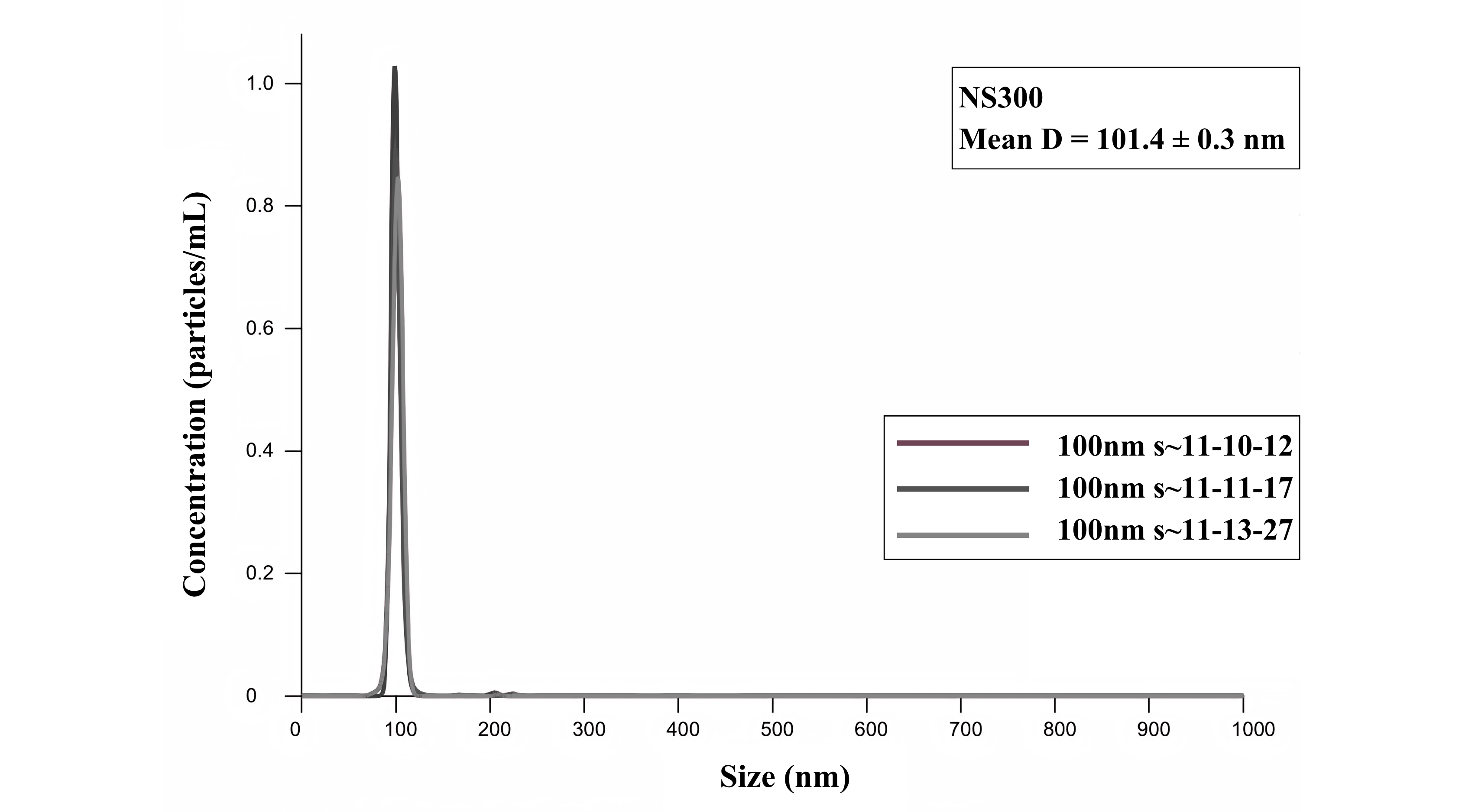

Supplement: Supplemental 1a [file jbt_2026_37_2_163225_348893.png]

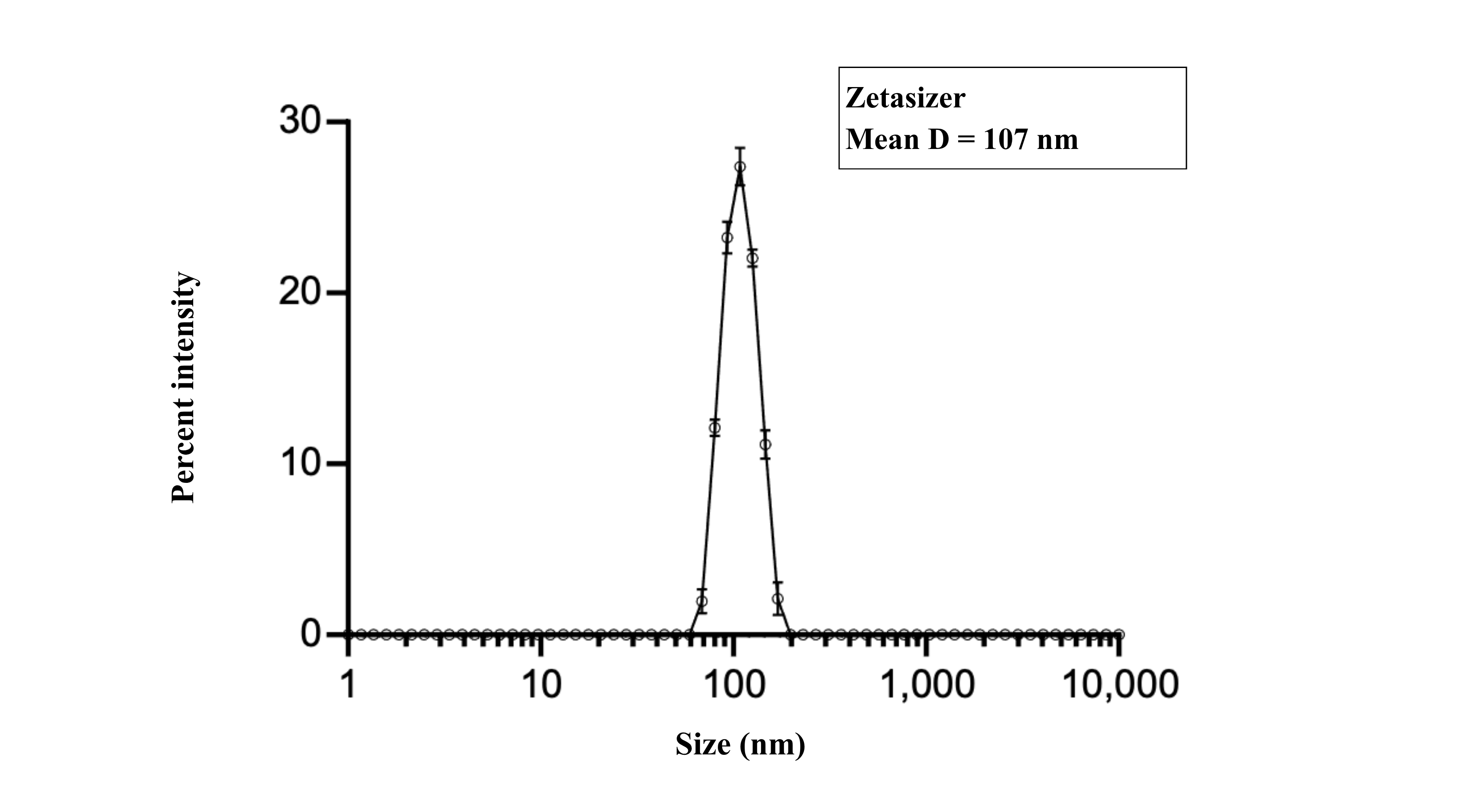

Supplement: Supplemental 1b [file jbt_2026_37_2_163225_348890.png]

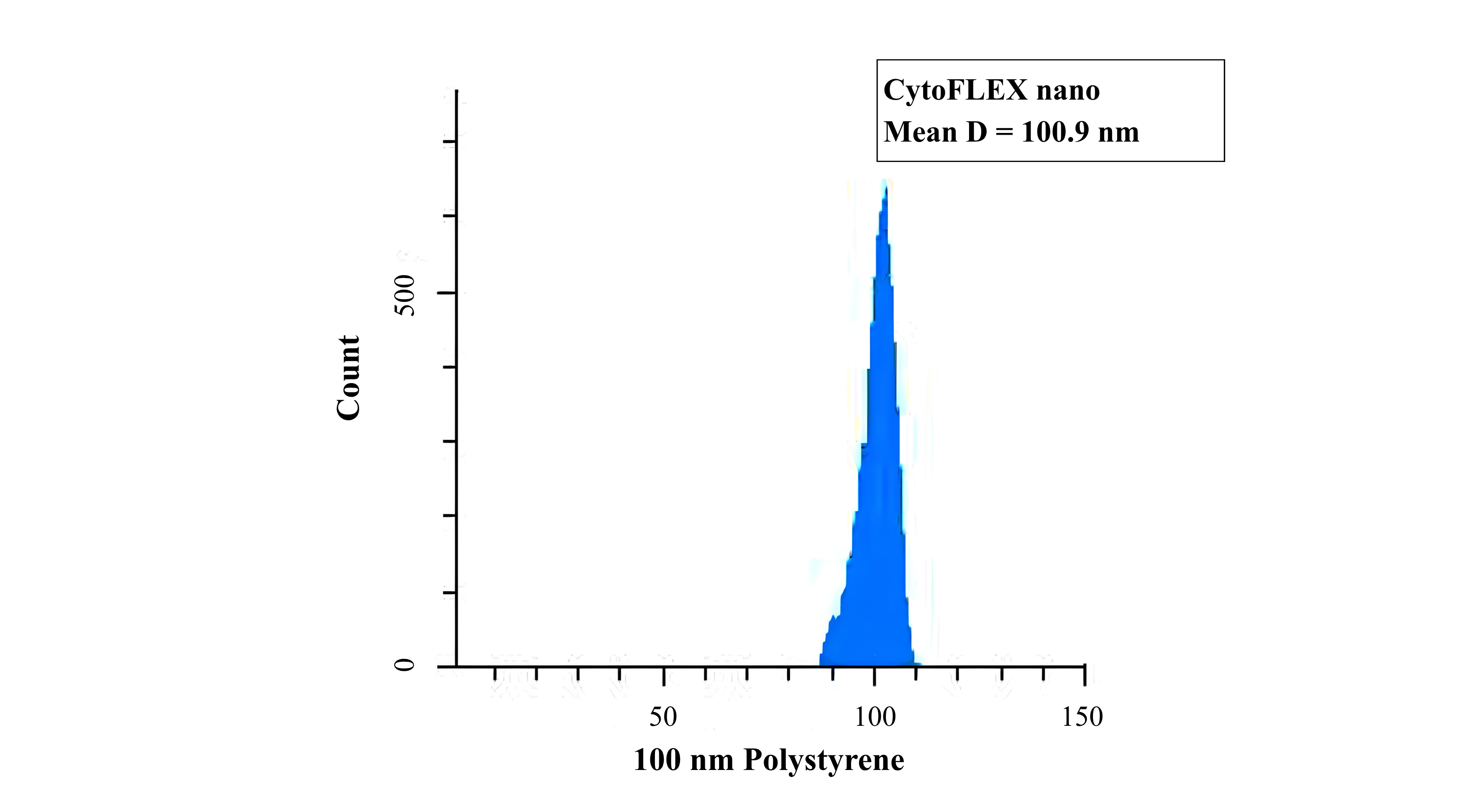

Supplement: Supplemental 1c [file jbt_2026_37_2_163225_348887.png]

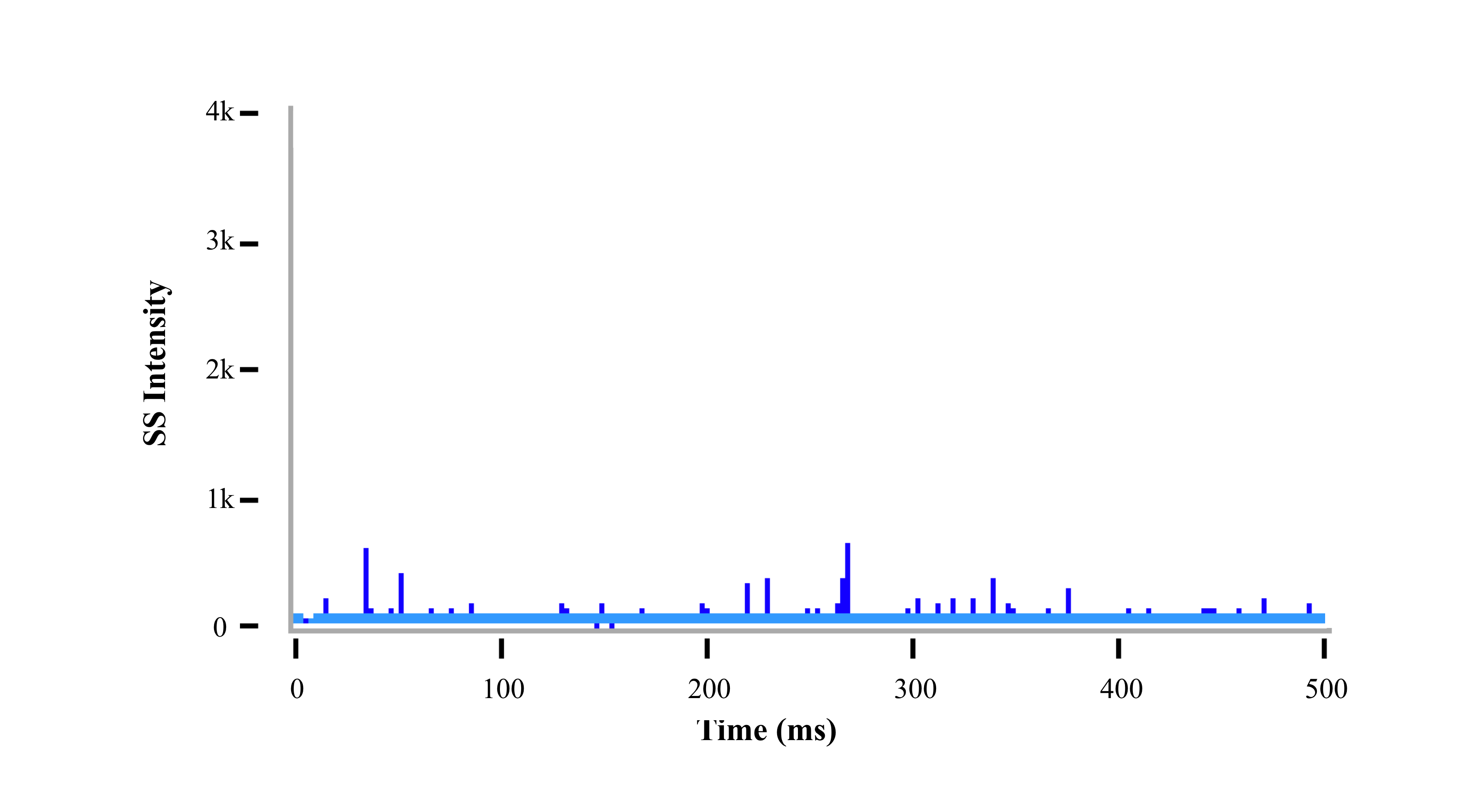

Supplement: Supplemental 2 [file jbt_2026_37_2_163225_348888.png]

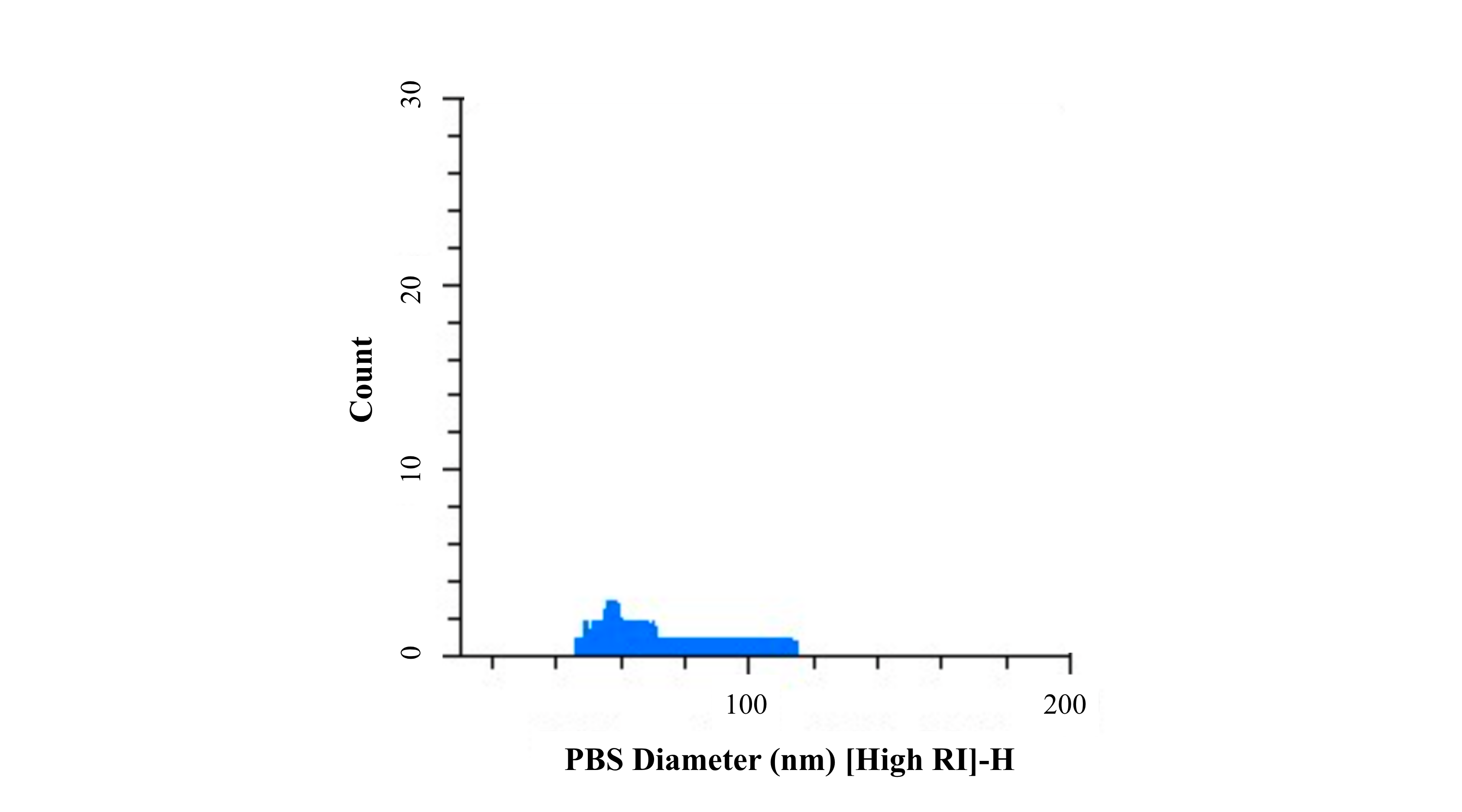

Supplement: Supplemental 3 [file jbt_2026_37_2_163225_348889.png]

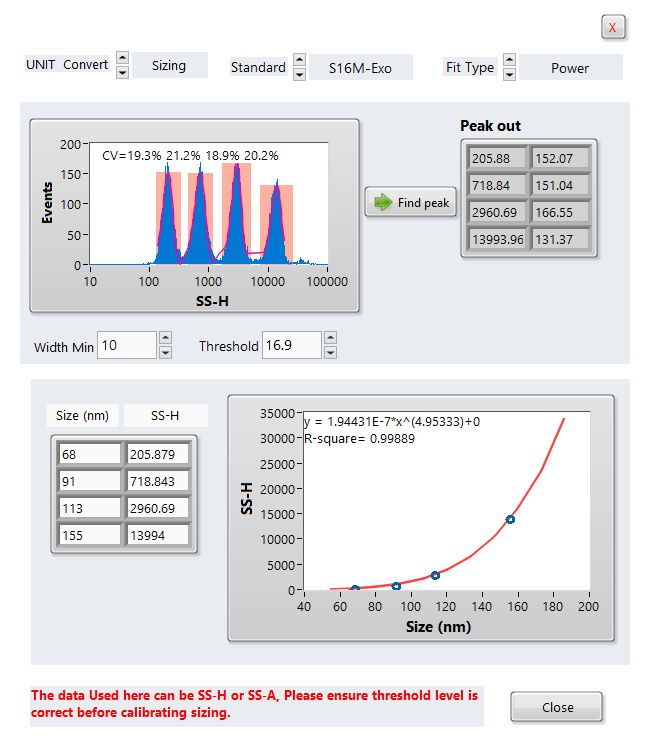

Supplement: Supplemental 4 [file jbt_2026_37_2_163225_348891.png]

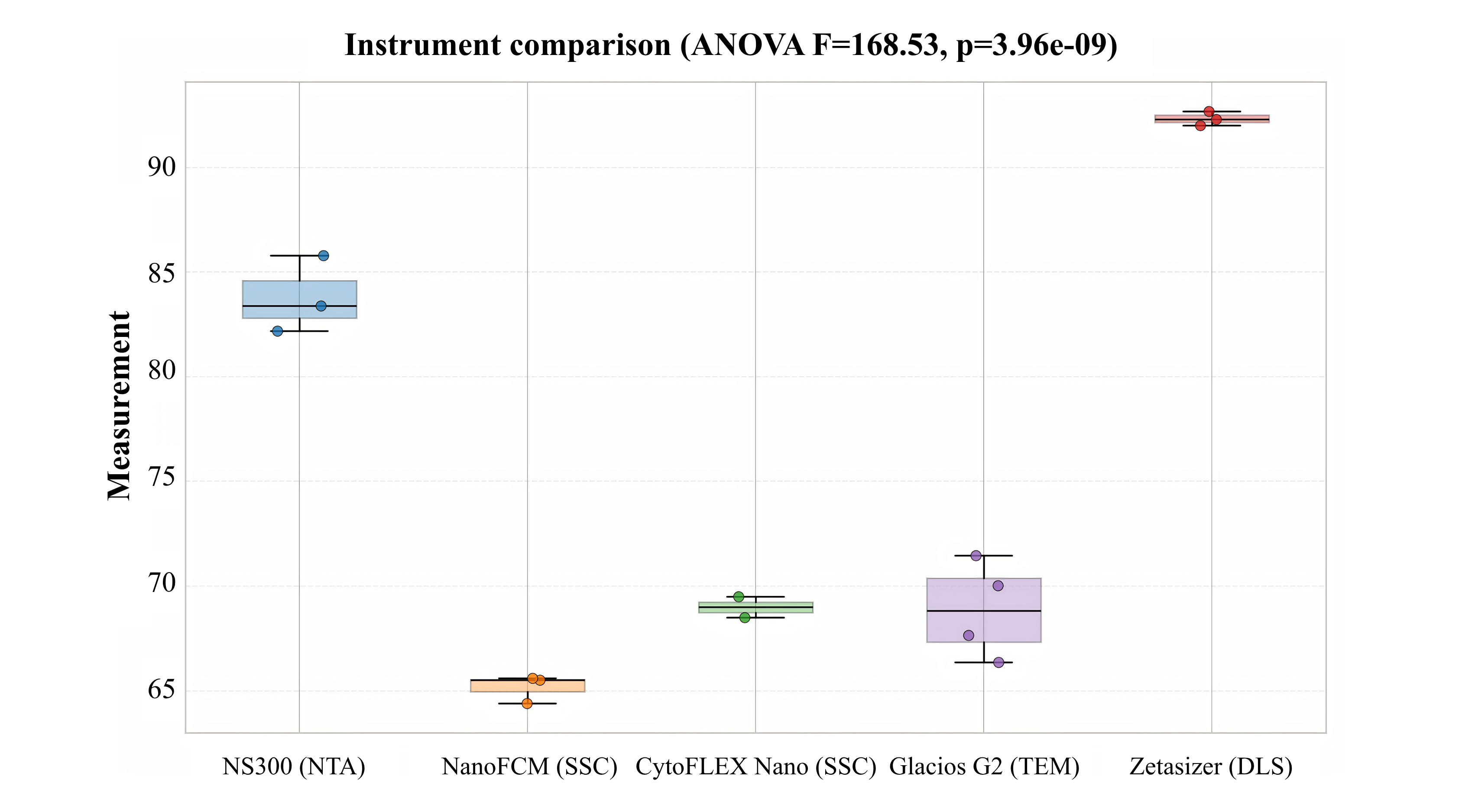

Supplement: Supplemental 5 [file jbt_2026_37_2_163225_348892.png]
